# Two Methods of Monitoring Cats at a Landscape-Scale

**DOI:** 10.3390/ani11123562

**Published:** 2021-12-15

**Authors:** Cheryl A. Lohr, Kristen Nilsson, Ashleigh Johnson, Neil Hamilton, Mike Onus, Dave Algar

**Affiliations:** Biodiversity Conservation Science, Department of Biodiversity, Conservation and Attractions, 17 Dick Perry Avenue, Kensington, WA 6151, Australia; kristen.nilsson@dbca.wa.gov.au (K.N.); ashleigh.johnson@dbca.wa.gov.au (A.J.); neil.hamilton@dbca.wa.gov.au (N.H.); mike.onus@dbca.wa.gov.au (M.O.); dave.algar@dbca.wa.gov.au (D.A.)

**Keywords:** feral cat, *Felis catus*, Australia, Indigenous Protected Area, 1080

## Abstract

**Simple Summary:**

Feral cats are difficult to manage and harder to monitor. We report on the efficacy of *Eradicat^®^* baiting and the cost and the efficacy of monitoring the activty of feral cats via camera-traps or track counts. Pre-baiting surveys for 2020 and 2021 suggested that the population of feral cats on Matuwa was very low, at 5.5 and 4.4 cats/100 km respectively, which is well below our target threshold of 10 cats/100 km. Post-baiting surveys then recorded 3.6 and 3.0 cats/100 km respectively, which still equates to a 35% and 32% reduction in cat activity despite initial low cat detection rate. Track counts recorded more feral cats than camera traps and were cheaper to implement.

**Abstract:**

Feral cats are difficult to manage and harder to monitor. We analysed the cost and the efficacy of monitoring the pre- and post-bait abundance of feral cats via camera-traps or track counts using four years of data from the Matuwa Indigenous Protected Area. Additionally, we report on the recovery of the feral cat population and the efficacy of subsequent *Eradicat^®^* aerial baiting programs following 12 months of intensive feral cat control in 2019. Significantly fewer cats were captured in 2020 (*n* = 8) compared to 2019 (*n* = 126). Pre-baiting surveys for 2020 and 2021 suggested that the population of feral cats on Matuwa was very low, at 5.5 and 4.4 cats/100 km, respectively, which is well below our target threshold of 10 cats/100 km. Post-baiting surveys then recorded 3.6 and 3.0 cats/100 km, respectively, which still equates to a 35% and 32% reduction in cat activity. Track counts recorded significantly more feral cats than camera traps and were cheaper to implement. We recommend that at least two methods of monitoring cats be implemented to prevent erroneous conclusions.

## 1. Introduction

Feral cats (*Felis catus*), cats that live in the wild and can survive without human reliance or contact, are recognised as a key threatening process to native species in Australia [1,2,3] and around the world [4,5]. Predation by feral cats has been demonstrated to threaten the persistence of many native species [6,7], and causes billions of dollars damage to the natural and agricultural environment [8] alongside disease transmission [9]. Predation by feral cats has been identified as one of the major obstacles to the successful reintroduction of extirpated native fauna [10,11,12,13]. Therefore, the suppression of feral cat populations is a critical component to the successful conservation of small to medium-sized native fauna [14,15].

While implementing methods of feral cat control is difficult, measuring the outcomes of feral cat control, the pre- and post-management abundance of cats, is harder. Detecting feral cats is difficult because they are a cryptic species that avoids interactions with humans [16] and in some environments cannot be readily detected via remote sensing technologies, such as camera-traps, despite the use of lures [17,18]. Estimating the abundance of an animal species typically requires capturing or identifying individual animals on multiple occasions [19]. Capturing feral cats on multiple occasions is extremely difficult, requiring the use of multiple labour intensive techniques [20] and feral cats frequently lack the unique markings required to identify individuals for mark-resight analysis [21]. New analytical techniques such as N-mixture models and Royle–Nichols abundance models that may alleviate some of these issues require further investigation [22,23].

Feral cat control is primarily a task performed or funded by government agencies [1]. Between 1998 and 2003, $4.7 million was spent by Australian conservation organisations on labour associated with cat control with operational costs (materials, vehicles, equipment) requiring additional funding [24]. From an economic perspective, it is essential that we measure the outcomes of the pre- and post-management abundance of cats. Cats can breed rapidly, potentially doubling in abundance each year [25], and disperse over long distances [26,27]. This allows populations of feral cats to potentially recover quickly post-management. Moseby et al. [28] demonstrated that some fauna can survive and increase in abundance in the presence of a low density of cats (~0.5 km^2^). From a biological perspective, it is essential that consistent and ongoing monitoring of the pre- and post-management abundance of cats occurs to ensure we maintain a low density of cats. 

The challenge is to develop a cost-effective method of monitoring cats at a landscape-scale. Several recent studies have tested the efficacy of camera-traps with slight modifications to camera placement, lures, and survey duration with minor improvements in the efficacy of camera-traps [17,29,30,31]. Edwards et al. [32] compared track counts with spotlighting and concluded that track counts were a more reliable method of monitoring mammalian carnivores. These studies did not consider the cost of implementing these techniques. Lohr and Algar [33] used both camera-traps and track counts to monitor feral cats and concluded that camera-traps provide more reliable data, but are more expensive and time-consuming to implement than track counts. Track counts provide a cheaper, rapid survey technique, but are susceptible to error from inexperienced observers, and weather conditions erasing tracks. Lohr and Algar [33] did not formally analyse the cost or efficacy of these two techniques.

The purpose of this manuscript is to analyse the cost and the efficacy of monitoring the pre- and post-bait abundance of feral cats via camera-traps or track counts. Additionally, we report on the recovery of the feral cat population and the efficacy of subsequent *Eradicat^®^* aerial baiting programs on the Matuwa Indigenous Protected Area (IPA) following 12 months of intensive feral cat control [33] that consisted of aerial baiting and landscape-scale leg-hold trapping.

## 2. Materials and Methods

### 2.1. Study Site

**Figure 1:**
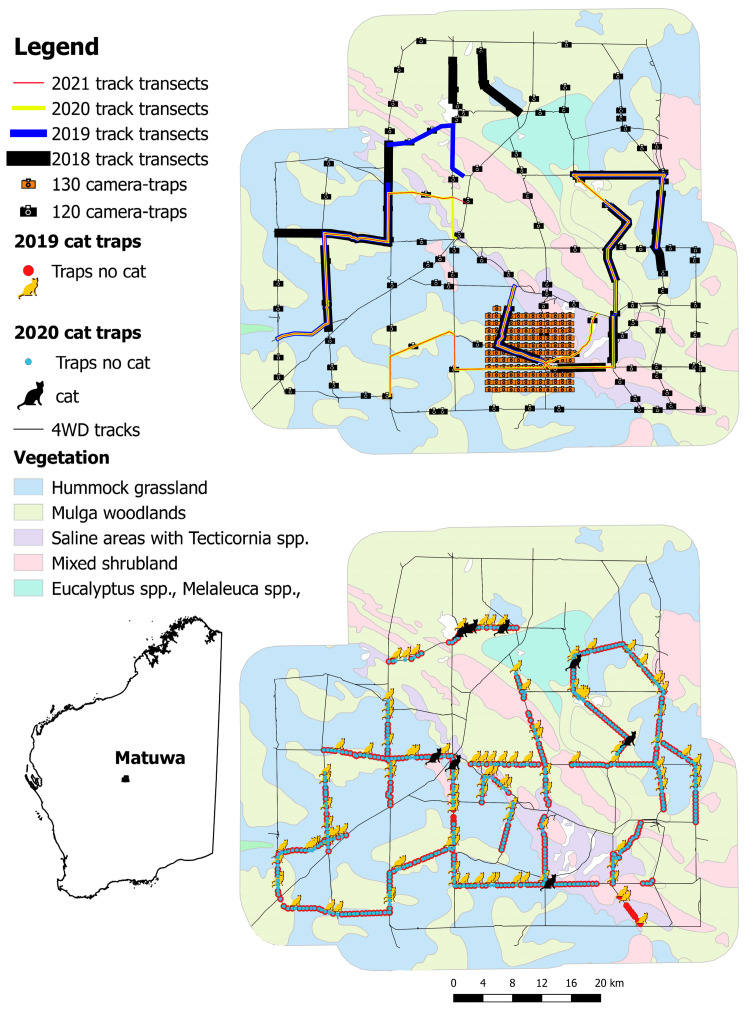
Maps of the feral cat monitoring (**top**) and trapping (**bottom right**) between 2018 and 2021 on the Matuwa Indigenous Protected Area in central Western Australia (**bottom left**).

The Rangelands Restoration program at the Matuwa Indigenous Protected Area (2440 km^2^; ex-Lorna Glen pastoral lease) in central Western Australia (26°13′ S, 121°33′ E; Figure 1) aims to achieve the successful reconstruction of an Australian arid zone native species assemblage. To date, five species have been successfully reintroduced to Matuwa; the bilby (*Macrotis lagortis*), common brushtail possum (*Trichosurus vulpecula hypoleucus*), Barrow Island golden bandicoot (*Isoodon auratus barrowensis*), burrowing bettong (*Bettongia leseuer*), and mala (*Lagorchestes hirsutus*), of which the final two are still confined to a predator-free fenced area [34,35,36,37]. The successful reintroduction of native species to the open landscape can only be maintained if an effective and sustained feral cat control program can be achieved [10,38,39,40].

Matuwa consists of two main land systems: (1) Bullimore—sand plains and dunes dominated by spinifex (*Triodia* spp.); and (2) Sherwood—breakaways and stony plains dominated by mulga and other acacia shrublands with the most common vegetation unit being mulga (*Acacia aneura*) and *Eucalyptus kingsmillii* over hummock grasslands (*Triodia basedowii*) [41,42]. Matuwa, being in the arid zone, is characterized by extreme temperatures and low and erratic rainfall with an annual average of 261.7 mm (Bureau of Meteorology, records 1898–2018; Wiluna weather station No. 13012 located 137 km WSW of Matuwa). Average maximum daily temperatures range from 19 °C in winter to 38 °C in summer, and average minimum temperatures range from 5 °C in winter to 23 °C in summer.

### 2.2. Feral Cat Management

Since 2003, we have been using the poison bait known as *Eradicat^®^* on Matuwa to control feral cats [38,43,44]. *Eradicat^®^* baits contain 4.5 mg of directly injected toxin ‘1080′ (sodium monofluoroacetate). Prior to being laid, feral cat baits are thawed in direct sunlight and sprayed, with an ant deterrent compound (Coopex^®^) at a concentration of 12.5 g/L. This process is aimed at reducing ant attack and maintaining the palatability of the bait to cats. Most years, baits are deployed from a fixed wing aircraft at a rate of 50 baits/km^2^ during the cool, dry winter periods when the abundance and activity of all prey types is at its lowest [44]. Since Matuwa is a site subject to adaptive management [33] there has been some variation in the portion of Matuwa subject to aerial baiting and/or ground based baiting. Hence, for this analysis, we have selected data from feral cat survey points that occurred on portions of Matuwa subject to aerial baiting only between 2018 and 2021 (minimum 1353 km^2^).

Three feral cat trapping programs were conducted at Matuwa between 2018–2020. The first, a small-scale exercise with 1600 trap-nights, was conducted immediately following the baiting program in August 2018 to provide a snapshot of the population demographic of resident cats that had survived the baiting program [33]. The second, a more comprehensive trapping program with the goal of reducing the abundance of feral cats on Matuwa and a total of 5398 trap-nights were conducted across the site prior to the baiting program in March and April 2019 [33]. The third trapping program, which is first published here, was conducted from 5 August to 6 September 2020, with each trap in commission for 10 consecutive days as per previous years (Figure 1). The whole trapping circuit, comprised of a linear track length of 280 km. The three trapping programs used the same personnel and trapping methodology. Trapping was conducted using pairs of padded leg-hold traps, Victor ‘Soft Catch’^®^ traps No. 1.5 (Woodstream Corp., Lititz, PA, USA), using a mixture of cat urine and faeces as the attractant. Trap pan tension was maintained at manufacturer standard to ensure that smaller cats were not excluded from the study thereby reducing the risk of biased demographic data. Trap locations recorded in 2019, using a Garmin GPS Rhino 650 (Garmin Ltd., Olathe, KS, USA), were used to re-position trap-sets in 2020. Of the 573 trap-sets deployed in 2019, 558 trap-sets were recommissioned in 2020. Fifteen trap sites were discontinued because of access difficulty along one track in the southeast corner of the property (Figure 1). All traps were checked each morning within three hours of sunrise, and any trapped cats were euthanised using a 0.22 calibre rifle shot to the head at point blank range. Chi-squared tests were used to test whether there were significant differences in captures between the two trapping programs.

### 2.3. Feral Cat Demographics

All animals captured were weighed and sexed; a broad estimation of age (as either kitten, juvenile or adult) was recorded using weight as a proxy for age. The yearling weight and age classes adopted in the previous study (see Table 1; adapted from Jones and Coman [45]) were used to define the population age structure. The pregnancy status of females was determined by examining the uterine tissue for embryos. 

### 2.4. Camera-Trap Monitoring

Two camera-trap arrays were used to monitor feral cat activity on Matuwa pre- and post-management. In 2018–2020, 120 camera-traps (Reconyx Hyperfire PC900 Professional camera; Reconyx, WI, USA) were installed using a stratified-random design based on the 20 most common geological types in the Wiluna region [46]. The cameras were placed between 30 m and 200 m away from an ungazetted track (Figure 1). Camera-traps were, on average, 2.80 km from their nearest neighbour (min = 1.0 km, max = 5.9 km). Spatial autocorrelation is unlikely at this scale [47] despite the potentially large home-ranges of feral cats [48]. Data from 70 of these cameras that were placed in an aerial baited site in 2018 and 2019 were used in subsequent analysis [33]. In 2020 and 2021, the entirety of Matuwa was aerial baited. Data from all 120 cameras in 2020 were used. In 2020–2021, a grid of 130 camera-traps with cameras spaced 1 km apart was installed on Bullimore sandplain in the south of Matuwa (Figure 1). Previous research has shown that feral cats are most active on sandplains [33].

All cameras were mounted on a 30 cm high plastic sand peg, in a horizontal position, facing south, in a space with at least 3 m of open ground in front of the camera. Two olfactory lures (Canines-a-plenty and Catastrophic from Outfoxed Pest Control, Victoria, Australia) were placed on two natural sticks approximately 30 cm tall and 1 m apart, 3 m from the front of the camera and refreshed at least 10 days before and after management. Herbaceous vegetation immediately in front of the camera was removed. Camera-traps captured three photos per trigger, with no quiet period. Timed photos were taken at 23:00 h to monitor the operation of the camera. 

The efficacy of the lures is thought to fade over time. Therefore, we used data from the first 10 days after camera-traps and lures were set, pre- and post-management. Photos were stored in the Colorado Parks and Wildlife Photo Warehouse database (CPW) [49] and viewed by at least two observers to confirm species identification. A histogram of time intervals between consecutive photos revealed that 99.6% of photos were captured either <5 min apart or >60 min apart (Supplementary Materials, Figure S1). To minimise temporal autocorrelation, we grouped consecutive photos that were <5 min apart to create independent records for subsequent analysis [50].

### 2.5. Track Counts

Track counts collect data that reflects that activity of feral cats on unsealed roads referred to as the track activity index (TAI). Approximately two weeks pre- and post-baiting, two teams of experienced observers ran a single TAI transect at least 50 km in length each day [33,38] for four consecutive days (Figure 1). Teams alternated transects each day to reduce observer bias. Since Matuwa is a site subject to adaptive management [33] there has been some variation in the placement of TAI transects between years (Figure 1), but the placement of TAI transects is consistent within years pre- and post-cat management. 

TAI-transects occur on sandy 4WD tracks, which are initially cleared by towing a heavy iron drag behind a 4WD vehicle. Observers, driving all-terrain vehicles (ATVs) at a speed of 10–15 km/h then inspect the transect for cat tracks, and clear new signs of animal activity by towing a chain iron drag. Cat tracks that occur within 1 km radius of one another on a daily survey are aggregated into one cat detection to minimise spatial autocorrelation. A histogram of distances between consecutive cat tracks revealed that 92.3% of tracks were <1 km apart and usually caused by cats that travelled along roads leaving a continuous set of prints, which were documented as having left one discrete set of prints every 100 m for the purposes of this analysis only (Supplemental Information, Figure S1). The single 50 km transect is split at disused wells and intersections with any observations recorded within 1 km of the well or intersection being discarded. The number of cats observed on each TAI-transect is scaled against the total length of the TAI-transect within each day and then averaged across sequential survey days. Only TAI-transects that occurred on areas that were aerially baited with *Eradicat^®^* were analysed.

### 2.6. Analysis of Monitoring Data

Count data from camera-traps and continuous TAI data were analysed via negative binomial mixed-effects models with a parameter for zero-inflation in the R (V4.0.2, [51]) package *glmmTMB* [52], with monitoring method (dispersed camera-traps, grid camera-traps, or track count), survey (pre- or post-management and post-trapping), and year as factorial fixed effects, while year and TAI-transect name or camera ID were used as random effects. Models were generated through a backward stepwise refinement process after fitting a global model and reviewing the significance of individual fixed effects. We used the *DHARMa* package [53] to review model residuals and fit. Models were ultimately compared via Akaike’s Information Criterion (AICc) in the package *AICcmodavg* 2.3-1 [54].

## 3. Results

### 3.1. Feral Cat Demographics

The 2020 trapping program, which was conducted over 5539 trap-nights, resulted in the capture of eight cats (5 males, 3 females). A number of the 558 traps were decommissioned early when they captured non-target species, or when red kangaroos (*Osphranter rufus*) and euros (*O. robustus erubescens*) destroyed the trap-set. Percentage trap success was 0.14 cats/trap-night; capture locations are presented in Figure 1. No kittens or juvenile cats were captured. There was no significant difference between adult male and female captures (Chi^2^ = 0.5, df = 1, *p* > 0.50). Two of the adult males were 2+ years of age (weights 4.3 and 4.6 kg) and the remaining were adults 1–2 years of age with a mean weight of 3.6 (±S.E. 0.3) kg, range 3.1–3.9 kg. The three females captured were all adults 1–2 years of age with a mean weight of 2.8 (±S.E. 0.1) kg, range 2.8–2.9 kg. None of the trapped females were pregnant. 

**Figure 2:**
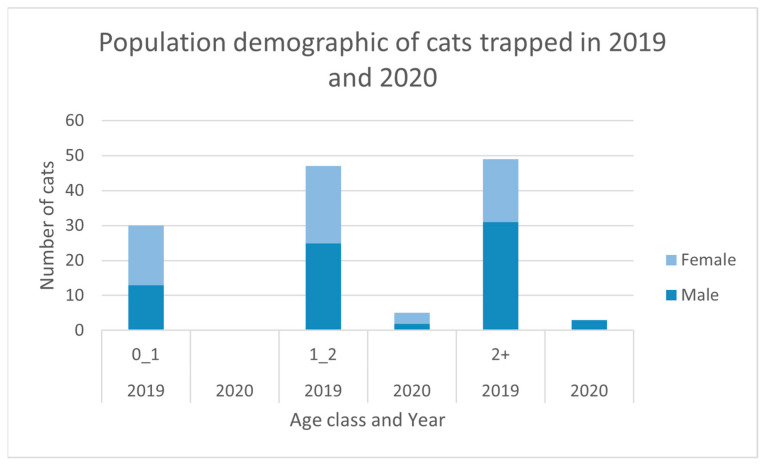
Sexes and age classes of the cats captured in 2019 and 2020.

Assuming equal trappability between years, there was a significant difference in the numbers of cats trapped with greater numbers in 2019 (*n* = 126) compared to 2020 (*n* = 8, Chi^2^ = 103.9, df = 1, *p* < 0.001), despite 141 fewer trap-nights (Figure 2). Similarly, more cats were trapped along the same trap-lines used during the pilot study of 2018 (*n* = 33) than at the same time of year in 2020 (*n* = 1). This was a significant difference (Chi^2^ = 20.17, df = 1, *p* < 0.001), with a percentage trap success of 1.87 cats/trap-night in 2018 compared to 0.08 cats/trap-night in 2020.

### 3.2. Monitoring Feral Cats

**Figure 3:**
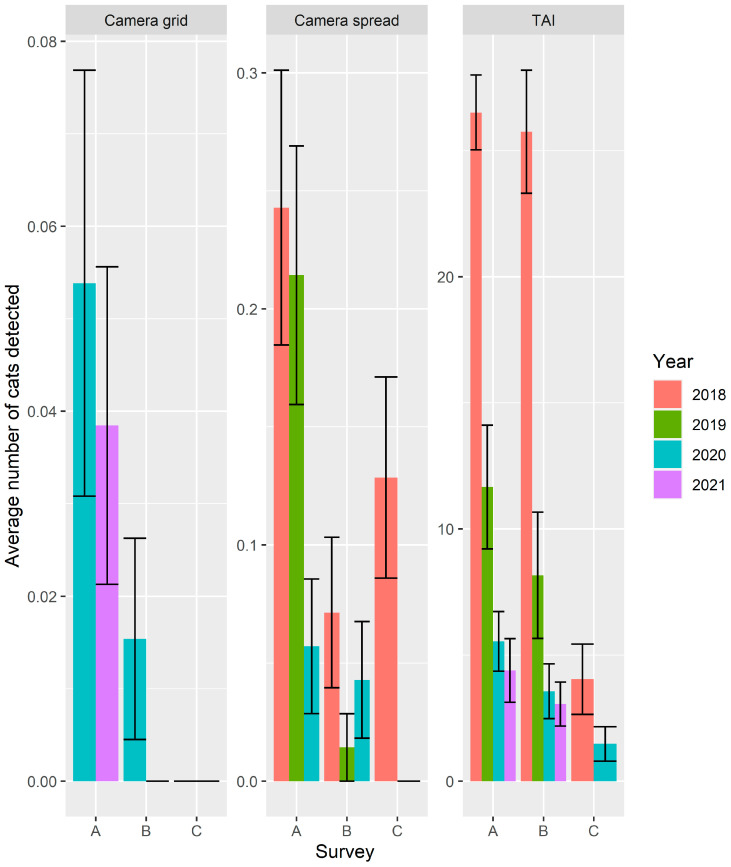
Average number of feral cats detected per survey on Matuwa via camera-traps in grid array (*n* = 130) or spread array (*n* = 120) and track counts (TAI-transects) with standard error bars. Survey notation: ‘A’ = pre-baiting survey; ‘B’ = post-baiting survey; ‘C’ = post-trapping survey, which occurred in August 2018 and August 2020.

The full suite of negative binomial mixed effects models revealed that the various arrangements of the cameras did not collect significantly different numbers of cat detections (*p* = 0.92; Model 1; Online Supplementary Materials). Therefore, through the iterative model refinement process we pooled data from the two camera arrays resulting in 120 cameras being deployed in 2018 and 2019, 250 cameras in 2020, and 130 cameras in 2021. We assessed the goodness-of-fit of the remaining models via R package *DHARMa* [53] and selected the Model 4 as the best model because the residuals did not significantly deviate from the expected distribution (Kolmogorov–Smirnov (KS) test = 0.58) nor were there significant outliers (*p* = 0.92; Table 2). The 130 cameras used in a grid array in 2021 were placed in spinifex sandplain habitat, which consistently recorded the greatest number of cats in prior studies [33]. From our best model (Table 2; Model 4) the TAI did record significantly more cat detections than cameras (*p* = 4.59^−15^; Figure 3). Pairwise comparisons revealed that the average number of feral cat detections during the pre-baiting survey was significantly higher than post-baiting surveys (*p* = 1.60^−9^) and post-trapping surveys (*P* = 2.02^−6^). The difference between post-baiting and post-trapping surveys was not significantly different (*p* = 0.93). In our second best model (Model 5, Table 2, KS = 0.72) year significantly affected the number of cats detected revealing that 2018 recorded significantly more cats than 2019 (*p* = 6.40^−4^), 2020 (*p* = 2.14^−13^) or 2021 (*p* = 1.35^−15^). This result confirms that the second comprehensive trapping program for feral cats in March and April 2019 (prior to pre-baiting survey of 2019) significantly reduced the on-going detection of feral cats on Matuwa. The proportion of camera-traps or TAI-transects that recorded zero cats was considerable with 65% of TAI transects, 99% of cameras spread across the landscape, and 99.8% of cameras in the grid recording zero cats each day.

In 2020 and 2021, pre-baiting surveys suggested that the population of feral cats on Matuwa was very low, at 5.5 (SE ± 1.2) and 4.4 (SE ± 1.3) cats/100 km, respectively, which is well below our target threshold of 10 cats/100 km [33]. Post-baiting surveys then recorded 3.6 (SE ± 1.1) and 3.0 (SE ± 0.9) cats/100 km, respectively, which still equates to a 35% and 32% reduction in cat activity in 2020 and 2021. Wide error margins around the average number of feral cats detected by 100 km of TAI transects is to be expected as the activity of feral cats varies with habitat type [33].

Considerably more cats were detected in 2018 prior to the second comprehensive trapping program for feral cats in March and April 2019 potentially inflating the statistical significance of year and survey. Re-analysing camera-trap and TAI data from 2020 and 2021 only, using model formulation 5 (KS = 0.41; Outlier test = 0.59) reveals ongoing significant difference between the pre-baiting and post-baiting surveys (*p* = 1.08^−4^; Figure 3), despite the initial low abundance of cats.

The camera-trap data was more severely zero-inflated resulting an average feral cat detection rate for track counts that was 5 to 25 times higher than the average detection rate for camera-traps. This difference may be masking the value of camera-trap data in statistical analysis. Re-analysing data from camera-traps and TAI-transects separately reveals that camera-trap data did not detect a significant difference between survey periods (*p* > 0.72) or years (*p* > 0.51), whereas TAI-transects did detect a significant difference between 2018 and subsequent years (*p* > 3.90^−5^), no difference between the pre-baiting and post-baiting surveys (*p* = 0.80) and a significant difference between pre-baiting and post-trapping surveys (*p* = 2.74^−3^; Figure 3; Online Supplemental Information). The 10-day camera-trap survey in the post-baiting period of 2021 detected zero cats, as did the post-trapping survey of 2020, which may lead to erroneous conclusions. The camera-trap data also suggest that the number of cat detections increased in 2018 following trapping, whereas track-counts suggested the opposite trend (Figure 3).

### 3.3. Cost

Feral cat monitoring on Matuwa is a long-established project. Table 3 illustrates the operational costs associated with initiating either TAI-transects or camera-trap monitoring on a new site for four years in 2021 with salary calculated as the average hourly rate of the people involved in monitoring on Matuwa. We do not include travel costs, analysis, or overtime benefits as these are site, project, and employee specific and hence are not costs that are transferable to other budgetary frameworks. We assume that a feral cat management project would only use one camera array. Ultimately, implementing camera-traps is twice as expensive as track counts for detecting feral cats (Table 3). 

## 4. Discussion

Track counts proved to be cheaper to implement and more effective at detecting feral cats, especially when cat density was very low. Feral cats typically have a low probability of detection per night by cameras [31], possibly because each camera has a field of view of 40° by 30.5 m [55] and hence surveys a maximum 320 m^2^. Reconyx cameras are designed to capture larger species such as deer (Cervidae) [55]. Feral cats are smaller and less likely to be detected when 30 m from the camera [56]. An array of 130 camera-traps used over 10 nights, surveys approximately 417,846 m^2^. In contrast, 100 km of track counts on 4WD tracks approximately 3 m wide over four nights, surveys approximately 1,200,000 m^2^.

On Matuwa, the low probability of detecting feral cats via cameras meant that during two surveys (post-trapping survey of 2020 and post-baiting period of 2021) zero cats were detected and therefore no estimate of cat activity or population density could be derived. Given that both surveys occurred after the implementation of feral cat management actions we could make a type 1 error by falsely concluding that we had removed all feral cats from the property. Use of a second method of detecting cats prevented that erroneous conclusion.

In 2018, data from camera-traps suggested that leg-hold trapping increased the number of feral cat detections and hence may have increased the abundance of feral cats on the property. Similarly, other studies have detected a net positive effect on cat detections following the use of toxic baits [57]. In eastern Australia, this nonsensical result was attributed to a significant reduction in cat activity at a paired unbaited site, which biased model outputs [57]. We attribute our increased number of feral cat detections to increased activity in feral cats, which were not removed by our management actions and may have been expanding their territories and seeking sign of conspecifics, as a result of neighbouring individuals being removed and the use of scent-based lures at camera-traps. The use of a second method of detecting cats prevented us from drawing a flawed conclusion from our 2018 data. No lures are used during track counts. 

The use of multiple monitoring systems for feral cats is advisable, particularly in eradication programs either on islands or within fenced areas, especially as the population declines to low numbers. Multiple monitoring systems were crucial during the eradication of feral cats from Dirk Hartog Island off the coast of Western Australia where the last cat was not recorded on camera, but its sign was observed on a track transect [58]. Evidence of this cat sign resulted in deployment of traps in that area and the subsequent removal of the last cat. Where the soil substrate is not sand, and track counts are difficult to implement, alternative monitoring techniques, such as importing sand to make sandpads [24,59] and/or deployment of hair snares [60], could be used to complement and verify data from camera-traps. 

During trapping in 2020, no cats in the 0 to 1 age class were captured suggesting that the baiting program in 2020 had a significant impact on this age class. Prior studies have concluded that juvenile and female cats are more susceptible to toxic baits as they have higher energy requirements [33]. Our data seem to support this hypothesis. Future research should continue to monitor the demographics of the feral cat population because consistent loss of juveniles to baiting would confirm that any recovery in the feral cat population at Matuwa is a result of immigration from neighbouring properties.

Ecosystems are spatially and temporally dynamic. The abundance of fauna is a response to dynamic abiotic (e.g., rainfall) and biotic (e.g., abundance of food) conditions. It is difficult to ensure that an experimental design, with sufficient power, replicates and control treatments, is applied to research that occurs at a landscape-scale [61]. We used a study design that consists of repeated before/after sampling at a single site [62]. Two potential control sites for Matuwa were discarded: Kurrara Kurrara, a property to the north, has considerably more salt lake country, which feral cats rarely use [33], and considerably more feral herbivores [63]; Jundee, a property to the west, has a similar habitat assemblage, but is managed as a pastoral lease and mine site and hence offers potentially confounding variables at a landscape-scale. Likewise, other neighbouring properties that should experience sufficiently similar climatic variables are functioning cattle stations with potentially confounding variables. Sampling designs that use only a single control site to contrast against a single potentially impacted site (BACI) may be confounded with any pre-existing cause of variability between the two locations [62]. Comparing a single year of *Eradicat^®^* baiting in a National Park to a cattle station with only 30 replicates for calculating a relative abundance of cats is inappropriate, as it is unlikely to be statistically robust given the variation in management regimes [64]. If typical climatic conditions are maintained throughout the experiment, a large number of survey replicates are implemented, multiple monitoring techniques are used, and researchers carefully consider the ecological relationships among species, then reasonable inferences can be drawn from repeated before/after sampling at a single site with a pulse disturbance [62] such as baiting. 

We caution researchers against the use of single-season before/after sampling experiments without careful consideration of relevant ecological relationships and wider temporal and spatial context or the use of alternative methods of monitoring feral cats that may corroborate results. Wysong et al. [30], for example, concluded that annual aerial baiting for feral cats was more effective at reducing the wild dog population than the feral cat population, and that the reduction in wild dogs had allowed an increase in the abundance of kangaroos [30], but did not consider the variation in timing of breeding behaviour and response to environmental conditions that these species may exhibit when interpreting their results. They could have used data presented in their other work [65] to assess the validity of their results. Two survey methods, track counts [66] and mortality of cats with GPS collars [65], concluded that aerial baiting reduced relative cat abundance by 61% [66] and 66% [65], whereas camera-traps and occupancy analysis found an approximately 15% reduction in relative cat abundance [30].

## 5. Conclusions

After many years of research, we conclude that feral cats are difficult to manage and harder to monitor. We recommend that at least two methods of monitoring cats be implemented to prevent inaccurate conclusions. Ideally, BACI designs are likely to be more informative where multiple control sites with a similar management history are available. We recommend an integrated pest management framework in designing feral cat management programs using both toxic baits and trapping/control methods.

## Figures and Tables

**Table 1:** The weight groups for the cat age classes of the trapped population.

Category	Male	Female
Kitten	<1.0 kg	<1.0 kg
Juvenile	1.0 < 3.0 kg	1.0 < 2.5 kg
Adult 1–2 years of age	3.0–4.0 kg	2.5–3.0 kg
Adult > 2 years of age	4.0+ kg	3.0+ kg

**Table 2:** Correct Akaike’s Information Criterion (AICc) results for zero-inflated negative binomial models of cat detections by camera-traps and track counts at Matuwa between 2018 and 2021. P-values associated with Kolmogorov–Smirnov test and outliers test in package DHARMa are included. The full suite of models are available in online Supplementary Materials. Model parameters are: I = integer count of cat detections; M = pooled methods of detection (camera-trap or track count); m = unpooled methods of detection (dispersed camera-traps, grid camera-traps, or track count); S = survey period (pre-baiting, post-baiting, or post-trapping); Y = Year; L = Location (camera ID or TAI-transect). Parameters in brackets are random effects. All models include a zero-inflation parameter.

Model	Parameters	K	AICc	Delta_AICc	KS	Outliers
1	I ~ M + S + Y + (1|L)	10	3488.28	0	0.59	7.71^−3^
2	I ~ M + S + (1|L)	7	3511.26	22.98	0.5	0.03
3	I ~ M + S + Y + (1|Y/L)	10	3525.68	37.4	0.07	0.02
4	I ~ M + S + (1|Y) + (1|L)	7	3676.17	187.89	0.58	0.92
5	I ~ M + S + Y	9	3817.38	329.1	0.72	0.62

**Table 3:** Operational costs of monitoring feral cats in Australian dollars.

Method	Item	# Units	$/Unit	Total $
Camera-traps	Reconyx cameras and accessories	125	1000	125,000
	Lure	8	35	280
	Field work, 8 surveys, 14 trips to install/remove of cameras, 5 days, 2 people	1120	48	53,760
	Photo ID, 8 surveys, 6.5 days, 1 person	52	48	2496
**Total**				**181,536**
TAI-transect	ATV	2	15,000	30,000
	Heavy and light drag	2	400	800
	Field work, 8 surveys, 5 days, 4 people	1280	48	61,440
	Data curation, 8 surveys, 1 h, 1 person	8	48	384
**Total**				**92,624**

## Data Availability

Data used in this study are available for download on Dryad (“Detections of feral cats on Matuwa between 2018 and 2021”; https://datadryad.org/stash/share/oKz1euLsTI10xU6RuN-8D-_j-PbLKpi_Jd6Sm0MdmkQ (accessed on 13 November 2021)).

## Supplementary Materials

The following are available online at https://www.mdpi.com/article/10.3390/ani11123562/s1, Figure S1: Histograms of the time interval or distance between cat detections on Matuwa with function facet zoom from package *ggforce* used to highlight the pattern in the data. (A) photos from 120 camera-traps spaced using a stratified random design and installed between 2018 and 2020, 96.7% of photos are <5 min apart. (B) photos from 130 camera-traps spaced using 1 km grid design and installed in 2020 and 2021, 99.5% of photos are <5 min apart. (C) distance between sets of cat tracks, 92.3% of tracks are <1 km apart and usually caused by cats that travelled along roads leaving a continuous set of prints, which were documented has having left one discrete set of prints every 100 m for the purposes of this analysis., Supplemental data T1: Negative binomial modelling results from analysis of camera-trap data and TAI-transects data.
