# Supplementary material for: Two Methods of Monitoring Cats at a Landscape-Scale
Source: Animals (Basel). 2021 Dec 15;11(12):3562. doi: 10.3390/ani11123562 (PMC8698172; doi:10.3390/ani11123562)
Supplement: Supplementary file 1 [file animals-11-03562-s001.zip › Supplemental information Fig 1 Histograms of detections.pptx]

## Slide 1
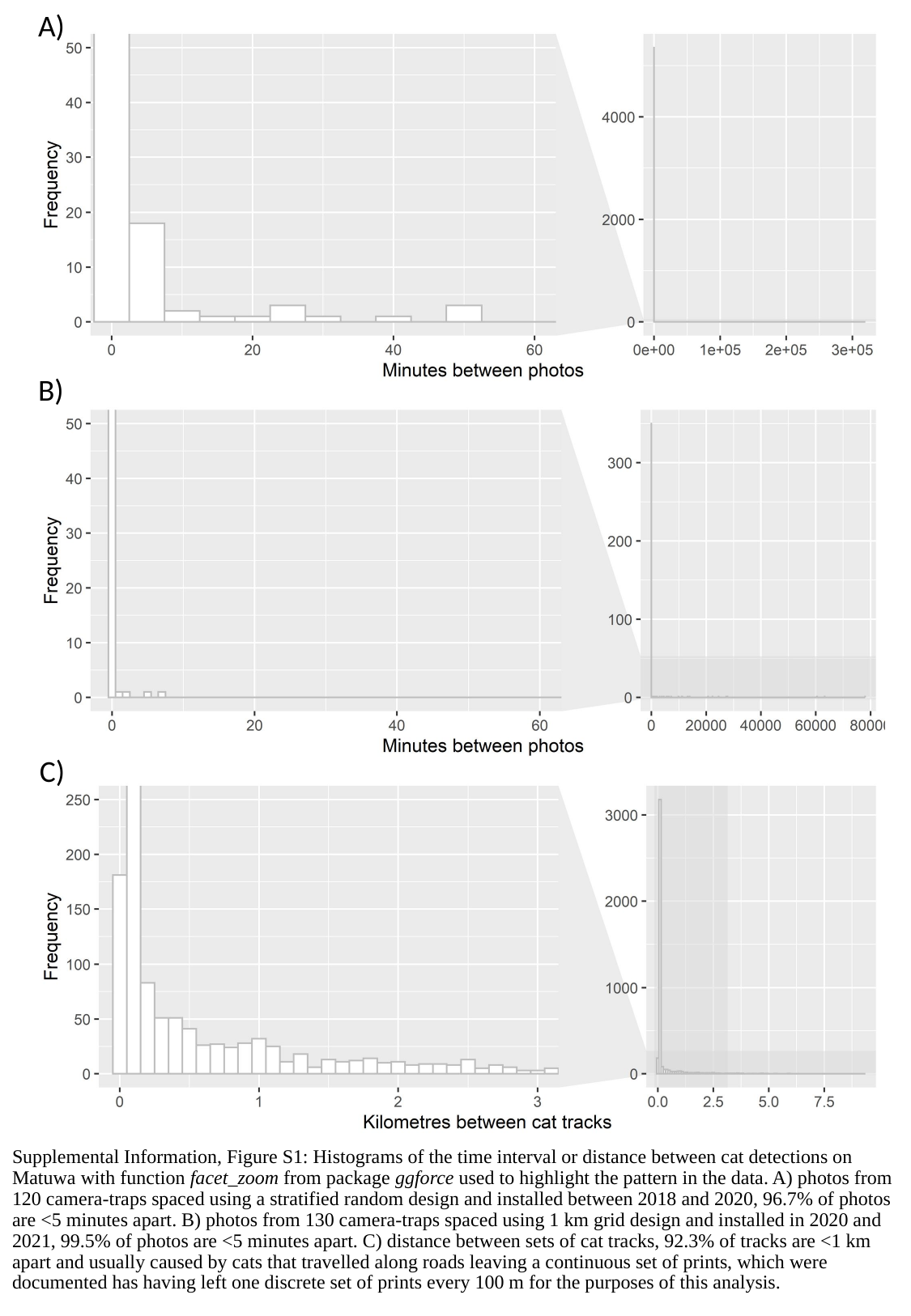

A)
B)
C)
Supplemental Information, Figure S1: Histograms of the time interval or distance between cat detections on Matuwa with function facet_zoom from package ggforce used to highlight the pattern in the data. A) photos from 120 camera-traps spaced using a stratified random design and installed between 2018 and 2020, 96.7% of photos are <5 minutes apart. B) photos from 130 camera-traps spaced using 1 km grid design and installed in 2020 and 2021, 99.5% of photos are <5 minutes apart. C) distance between sets of cat tracks, 92.3% of tracks are <1 km apart and usually caused by cats that travelled along roads leaving a continuous set of prints, which were documented has having left one discrete set of prints every 100 m for the purposes of this analysis.
